# Kaposi’s Sarcoma-Associated Herpesvirus and Host Interaction by the Complement System

**DOI:** 10.3390/pathogens9040260

**Published:** 2020-04-03

**Authors:** Seung-Min Yoo, Myung-Shin Lee

**Affiliations:** Department of Microbiology and Immunology, Eulji University School of Medicine, Daejeon 34824, Korea; smyoo@eulji.ac.kr

**Keywords:** KSHV, complement system, innate immunity, virus–host interaction, membrane attack complex

## Abstract

Kaposi’s sarcoma-associated herpesvirus (KSHV) modulates the immune response to allow the virus to establish persistent infection in the host and facilitate the development of KSHV-associated cancer. The complement system has a central role in the defense against pathogens. Hence, KSHV has adopted an evasion strategy for complement attack using the viral protein encoded by KSHV open reading frame 4. However, despite this defense mechanism, the complement system appears to become activated in KSHV-infected cells as well as in the region surrounding Kaposi’s sarcoma tumors. Given that the complement system can affect cell fate as well as the inflammatory microenvironment, complement activation is likely associated with KSHV pathogenesis. A better understanding of the interplay between KSHV and the complement system may, therefore, translate into the development of novel therapeutic interventions for KSHV-associated tumors. In this review, the mechanisms and functions of complement activation in KSHV-infected cells are discussed.

## 1. Introduction

Kaposi’s sarcoma-associated herpesvirus (KSHV), also known as human herpesvirus 8 (HHV-8), is the etiologic agent of Kaposi’s sarcoma (KS), primary effusion lymphoma (PEL), and multicentric Castleman’s disease (MCD) [1,2,3]. The incidence of KS is correlated with immune suppression. Indeed, KS occurs more often in acquired immunodeficiency syndrome (AIDS), whereas recovery from immunosuppression reduces the incidence of KS. PEL and MCD are also associated with immunosuppression [4]. However, considering that not all AIDS patients with severe immunosuppressive conditions develop KS, additional factors also appear to be involved in KS development [5]. Although the pathogenesis of KS remains unclear, a chronic inflammatory response with persistent viral infection is necessary for its development [6]. Moreover, the interplay between the virus and immune responses during KSHV infection significantly impacts the clinical outcomes of KSHV-associated disease.

The complement system is a critical mediator of innate and acquired immunity; thus, it contributes substantially to pathogen clearance from the host, while also contributing to the pathogenesis of many chronic immune and inflammatory diseases. However, during viral infections, the complement system not only exhibits antiviral effects but also plays pathogenic roles in disease progression [7,8,9,10]. Although the association of the complement system with the pathogenesis of KSHV infection has been recognized [11], this area of research has received only minimal attention thus far. Hence, the function and activation mechanisms of the complement system in the pathogenesis of KSHV infection remain poorly understood. It is, therefore, essential to advance these areas of research to inform the development of novel approaches for KSHV prevention and treatment. Here, we have discussed (1) the complement system, (2) the evasion of the complement system by KSHV, (3) the activation of the complement system in KSHV-infected cells, and (4) the role of the complement system in KSHV infection. 

## 2. Complement System

**Figure 1:**
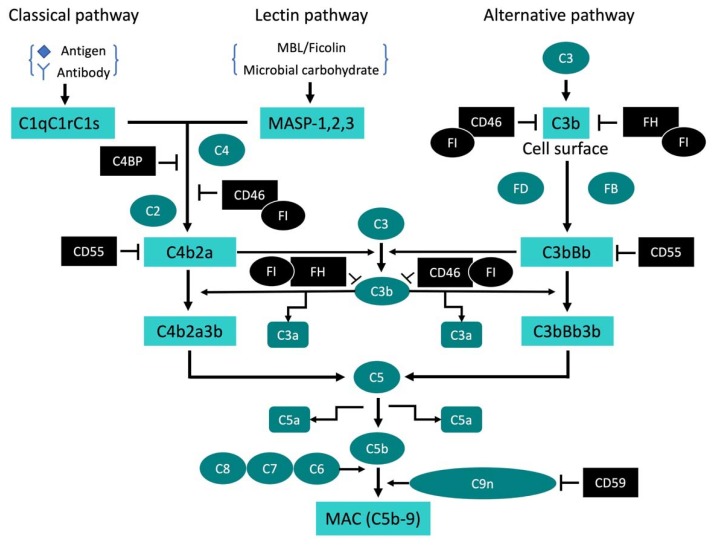
The three activation pathways of the complement system. All three pathways are activated by a different initiator; however, they share downstream C3 activation. C5b-9, also known as a membrane attack complex (MAC), is the final product of complement activation. The factor I (FI) works together with binding proteins, such as factor H (FH) and CD46. Black proteins: complement regulatory proteins that inhibit complement activation. FB: Factor B, FD: Factor D.

The complement system functions as the first line of defense against invading pathogens and is activated via three well-described pathways: classical, alternative, and lectin [12]. All three pathways merge to induce the activation of C3, which is followed by the formation of the membrane attack complex (MAC) (Figure 1). 

The classical pathway is activated by the antigen–antibody complex [13]. The first component of this pathway consists of three separate proteins, C1q, C1r, and C1s, which bind to the Fc portion of the antibody as the C1 trimolecular complex [14]. Since the C1 complex is held together by calcium-dependent bonds, it dissociates in the presence of calcium-chelating agents such as EDTA [15]. The active C1 complex recruits and cleaves C4 and C2, generating C3 convertase C4bC2a, which can activate C3 [12]. C3 is one of the most critical components in the complement system with the highest serum concentration [16]. For the complement cascade to proceed, C3 must be cleaved to C3a and C3b. C3b binds to the surface of cells or pathogens and acts as the initiator of the terminal complement pathway [12]. 

The lectin pathway is homologous to the classical pathway; however, it is activated by mannose-binding lectin (MBL) or ficolin, as well as several MBL-associated serine proteases (MASPs), rather than the C1 complex [17]. MBL and ficolin ligands are abundant in the cell walls of various pathogens, including bacteria, yeast, fungi, and viruses [17,18,19]. Binding of MBL or ficolin to its ligands activates the MASPs, which then induce C4 and C2 cleavage, leading to C3 activation in the same manner as that described for the classical pathway. 

The alternative pathway differs from the classical and lectin pathways in that it proceeds via trigger-factor-independent activation of complement on the cell surface [20]. Native C3 undergoes spontaneous hydrolysis in the serum, resulting in the continuous generation of low levels of C3b in the serum [20]. The C3b can bind to nearby cell surfaces in the absence of antibodies. This cell-bound C3b is short-lived and can either be inactivated by a regulatory mechanism involving several inhibitors or initiate the alternative pathway via interactions with factor B, factor D, and properdin. To prevent complement activation, the human body has several soluble and cell-bound regulatory proteins, such as factor H, CD46, CD55, and CD59 [21,22]. As factor H can bind to sialic acid and other neural or anionic polysaccharides on the cell surface, it can interact with glycoproteins on most mammalian cell surfaces [22]. C3b-bound factor H modifies the structure of C3b on the cell surface, causing it to become susceptible to an enzyme, factor I. Factors H and I destroy C3b as it is generated, resulting in the continuous inactivation of the alternative pathway [12]. However, without the intervention of factor H, factor B binds C3b on the cell surface. The bound factor B is, in turn, cleaved by a plasma serine protease, factor D, generating the Bb fragment that remains attached to C3b. The C3bBb complex works as a C3 convertase. The formation of the complex between C3b and factor B is magnesium-dependent; therefore, the alternative pathway is inactive in the absence of magnesium ion [23]. Activation of the alternative pathway readily occurs on microbial cell surfaces owing to the lack of regulatory proteins. However, in the plasma, properdin, a soluble glycoprotein, is a positive regulator of complement activation. The binding of properdin to C3b promotes the association of C3b with factor B, leading to formation of the C3 convertase (C3bBb) on the cell surface. Properdin can also bind, and subsequently stabilize, C3 convertase in the alternative pathway [22]. Studies have shown that the alternative pathway is initiated by not only properdin but also various proteases, including thrombin and kallikrein [24,25,26]. 

The final step in all three complement pathways involves the activation of C3 convertase by C5 convertase, followed by the formation of MAC [12]. C5b binds to the cell surface and subsequently interacts with C7 and C8. The C5b678 complex induces the polymerization of C9 to form a tubular structure consisting of poly C9, which forms a transmembrane channel designated as C5b-9 or MAC. C9 is structurally homologous to perforin, and MAC is similar to membrane pores formed by perforin [27]. While a subset of Gram-negative bacteria is susceptible to killing by MAC pores, generally, Gram-positive bacteria are not directly killed by complement activation, owing to their thick cell walls or capsules [28]. Additionally, specific pathogens, including Gram-positive bacteria, have evolved mechanisms to evade complement attack, such as recruitment, or mimicking, of complement regulators, modulation or inhibition of complement proteins by direct contact, and inactivation of complement proteins by enzymatic degradation [29]. Since erythrocytes have a limited capacity to resist membrane penetration, hemolysis is readily observed following complement activation [30]. However, nucleated eukaryotic cells can avoid complement attack as the plasma membrane contains many factors that inactivate complement proteins [31,32,33]. Furthermore, eukaryotic cells can recover from a MAC attack by removing MAC from the cell surface by shedding or internalizing MAC-containing membrane vesicles after MAC formation. Therefore, the effects of MAC on eukaryotic nucleated cells have been frequently reported to be sublytic, which triggers diverse outcomes through an ion influx, including cell proliferation, inflammatory cytokine release, and the production of growth factors [31,32,34,35]; moreover, all of these may contribute to the pathogenesis of various diseases.

The principal functions of the complement system are chemotaxis, opsonization, lysis of target cells, and priming of the adaptive immune system [12]. Polymorphic leukocytes and macrophages express receptors for C3a and C5a. The binding of C3a or C5a to its receptor enhances adhesion molecule expression on phagocytes and priming to release proinflammatory molecules, such as reactive oxygen species, inflammatory cytokines, and enzymes. Notably, the interaction between C3a or C5a and its receptors on tissue mast cells and basophils induces the massive release of histamine and cytokines, causing an allergic or anaphylactic reaction [36,37]. Complement activation causes complement fragments to efficiently coat the activator surfaces of targets to enhance their recognition by phagocytes, a process known as opsonization. As a result, phagocytes efficiently bind the target to promote phagocytosis and the secretion of lysosomal enzymes. 

## 3. Evasion of the Complement System by KSHV

Certain virus-encoded proteins inhibit complement activation in various ways [38,39]. Herpesvirus saimiri and murine gammaherpesvirus 68 (MHV-68), which are both lymphotropic herpesviruses, share an open reading frame (ORF) that encodes a protein structurally related to host regulators of complement activation (RCA proteins) [40,41]. RCA proteins encoded by MHV-68 inhibit activation of the murine classical and alternative complement pathways via inhibition of C3 deposition [40]. KSHV also contains an ORF with 44–55% homology to RCA proteins, designated as KSHV ORF 4 or KSHV complement control protein (KCP) [42,43]. The KSHV ORF 4 is a 1650-nucleotide sequence expressed as both a membrane-bound and secreted protein via alternative splicing. KSHV ORF4 is a type 1 membrane protein of 550 amino acids with four complement control protein domains at the N-terminus as well as a transmembrane-spanning domain at the C-terminus that functions in the attachment of KSHV ORF4 to cell or viral surfaces. There is also an ST-rich region between the complement control protein domains and the transmembrane-spanning domain [44]. Three KSHV ORF4 isoforms containing four complement control protein domains can be expressed by alternative splicing in KSHV-infected PEL cell lines with lytic replication [45]. When expressed, the 56 KDa KSHV ORF4 proteins from the yeast expression system inhibited the alternative- and classical-pathway-mediated lysis of rabbit or sheep erythrocytes in a hemolytic assay as well as C3 deposition on erythrocytes during complement activation [43]. In the same year, using a eukaryotic expression vector, another research group showed that the KSHV ORF4 expressed from CHO cells inhibited C3 deposition and complement-mediated hemolysis [42,45]. Inhibition of both classical- and alternative-pathway-mediated erythrocyte lysis was observed but to a lesser extent in the alternative pathway than in the classical pathway [42,43]. Mechanistically, KSHV ORF4 inhibits C3 convertases (C4b2a) in the classical pathway and acts as a cofactor for factor I-mediated cleavage of both C3b and C4b [46]. However, KSHV ORF4 is not only expressed in KSHV-infected cells but also located on the KSHV virion surface [45,47], which may serve as a defense mechanism for MAC damage in the KSHV envelope. Moreover, since the KSHV ORF4 has a binding affinity for heparin and heparan sulfate, it facilitates virus binding to host cells with other viral proteins such as gpK8.1. Antibodies against KSHV ORF4 were detected in KSHV-infected individuals; however, these antibodies did not inhibit the regulatory function of complement activation [48]. 

## 4. Activation of the Complement System in KSHV-Infected Cells

The complement system was highlighted in KSHV by studies that employed the KSHV ORF4; however, few studies have reported complement activation in KSHV-infected cells. A previous study found C3d and C5b-9 deposition in human KS tissue, especially around spindle cells [11]. In vitro studies also reported C5b-9 deposition in various KSHV-infected cells treated with normal human serum [49,50]. These results may provide evidence for KSHV infection serving as a trigger for complement activation. In this section, the current model for complement activation in KSHV-infected cells and their mechanisms will be discussed. 

### 4.1. Complement Activation in the KS Cellular Model 

Vascular endothelial cells are generally accepted as the origin of KS [51]. Like other herpesviruses, the life cycle of KSHV comprises latent and lytic stages. The virus first establishes a latent infection in cells for persistent infection; during this stage, viral gene expression is restricted to only a few genes. Conversely, during lytic replication, all viral genes are expressed, and the host cells are destroyed following the release of viral progeny [52]. Since lytic replication takes place in approximately 2% of spindle cells in KS tissue, a latent KSHV-infected cell model was initially preferred to investigate complement activation in KS-infected cells. In telomerase-immortalized microvascular endothelial cells (TIME cells), the addition of normal human serum did not induce complement activation [11]. Given that human vascular endothelial cells, including TIME and telomerase-immortalized umbilical vein endothelial (TIVE) cells, ubiquitously express CD46, CD55, and CD59 on their cell surface to inhibit complement activation [11,53], these results appear reasonable. Notably, the KSHV-infected TIME cells showed C5b-9 deposition following treatment with normal human serum. A similar result was detected in the long-term latently KSHV-infected TIVE cells (TIVE-LTC cells) [11]. While these results indicate that latently KSHV-infected cells activate the complement system, not all latently KSHV-infected cells can induce complement activation. 

**Figure 2:**
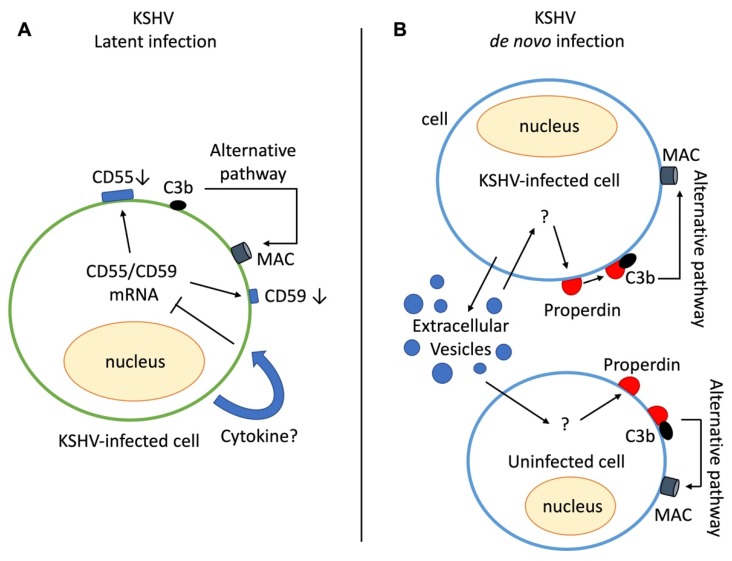
Activation mechanism for the complement system in Kaposi’s sarcoma-associated herpesvirus (KSHV)-infected cells. (**A**) The mechanisms for complement activation in latently KSHV-infected cells. Complement regulatory proteins are downregulated in latently KSHV-infected cells, which induce the alternative complement pathway. (**B**) The mechanisms for complement activation in de novo KSHV infection. In de novo infection of human endothelial cells, the complement regulatory factors are not suppressed. Extracellular vesicles from KSHV-infected cells serve as triggering factors for the deposition of endogenously produced properdin and C3 on the cell surface, which activate the alternative pathway. MAC: membrane attack complex.

In TIME and TIVE cells, the downregulation of CD55 and CD59 by KSHV infection serves as an inducible factor for complement activation [11] (Figure 2A). As mentioned above, CD55 and CD59 can inhibit complement activation by preventing the formation of C3 convertase and MAC, respectively (Figure 1). KSHV suppresses the mechanism underlying CD55 and CD59 expression, which might be a causative factor for the activation of the alternative pathway. However, the detailed mechanism underlying the suppression of CD55 and CD59 in KSHV-infected endothelial cells remains unclear. Cytokines may serve as regulatory factors for CD55 and CD59 expression. In fact, a previous study showed that TNF-α and IFN-γ decreased CD55 expression via the activation of ERK1/2 [54]. Furthermore, KSHV infection affects the production of cytokines in endothelial cells, which may be associated with the downregulation of CD55 and CD59. 

The analysis of complement activation in de novo KSHV-infected human umbilical cord vein endothelial cells (HUVEC) was more complicated than that in latently infected cells. KSHV infection in HUVECs induced lytic replication 48 h postinfection [55], resulting in an increased number of dead cells. Since apoptotic cells acquire the capacity to bind complement initiation molecules and lose complement regulatory proteins on the cell surface [56], C5b-9 can be deposited in KSHV-infected HUVECs by lytic replication. Interestingly, C5b-9 deposition was detected on KSHV-infected HUVECs even 24 h postinfection without lytic replication or apoptosis [49]. At this time point, apoptosis or cell death by lytic replication was not detected. Unlike latent KSHV-infected endothelial cells, there was no decrease in CD46, CD55, and CD59 in the de novo KSHV-infected HUVECs. The alternative pathway is also involved; however, here, the mechanism of complement activation appears different from that of the latently infected cells. Following KSHV infection in HUVECs, extracellular vesicles (EVs) from KSHV-infected HUVECs can activate the complement system in uninfected HUVECs. Since KSHV-infected HUVECs are affected by EVs via autocrine effects and the production of EVs cannot be completely abolished, it is difficult to determine whether other intracellular factors in KSHV-infected cells also contribute to complement system activation. Considering that the deposition of C5b-9 induced by EVs from KSHV-infected cells increases in a dose-dependent manner and that the suppression of EV production by GW4869 decreases complement activation, the EVs from KSHV-infected HUVECs likely contribute to complement activation [49]. Although the underlying mechanisms are unclear, properdin has been reported to be an initiation factor for the activation of the alternative pathway [57,58]. The properdin and C3 are expressed in human endothelial cells [59,60]. In EVs from KSHV-infected HUVECs, endogenously expressed properdin induced the deposition of C3b, followed by complement activation with normal human serum (Figure 2B). 

Factor H exhibits effects opposite to those of properdin in the activation of the alternative pathway [22]. As described earlier, Factor H is a negative regulator of the complement system and may have an important role in the complement activation of KSHV-infected cells. In a previous study, treatment of KSHV-infected TIME cells with factor H-depleted serum caused significantly higher levels of cell death than those observed in normal serum-treated cells [11]. Furthermore, colocalization of factor H and MAC formation was observed in KSHV-infected TIME cells [11]. Thus, factor H protects cells during complement activation, accounting for one of the survival mechanisms of KSHV-infected endothelial cells against complement attack. 

### 4.2. Complement Activation in Primary Effusion Lymphoma

PEL is a high-grade B-cell non-Hodgkin lymphoma with poor prognosis [61]. Some B-cell lymphomas are known to activate the complement system [62,63]. The two human gammaherpesviruses, Epstein Barr virus (EBV) and KSHV, are found together in approximately 80% of PEL cases [64]. EBV-infected B-cell lines activated the complement system more efficiently than EBV-negative cell lines [63]. Meanwhile, the KSHV-infected B-cell lines showed inconsistent results for complement activation [50]. BCP1, a B-cell lymphoma infected with KSHV but not co-infected with EBV, induced stronger C5b-9 deposition than a KSHV-negative B-cell lymphoma cell line via the activation of the alternative complement pathway. However, another KSHV-infected B-cell lymphoma cell line, BCBL-1, did not activate the complement system. Latently KSHV-infected human endothelial cells activate the alternative pathway via the downregulation of CD55 and CD59. While the KSHV-negative B-cell lymphoma cell line, BJAB, expressed CD55 and CD59, both the KSHV-infected PEL cell lines, BCBL-1 and BCP-1, did not express CD55 or CD59 [50]. This study suggests that KSHV may possess a suppressive mechanism to downregulate these complement regulatory proteins. Alternatively, CD46 was expressed in BCBL-1 cells, but not in BCP-1 cells. The expression of CD46 appears to determine the activation state of the complement system in KSHV-infected B-cell lymphoma. Moreover, both BCBL-1 and BCP-1 cells are positive for KSHV and negative for EBV. Therefore, CD46 appears to be regulated by cellular differences in each lymphoma cell line, rather than by infection with KSHV. 

## 5. The Role of the Complement System in KSHV-Infected Cells 

The complement system is considered to act as a sensor capable of differentiating between foreign, altered, and healthy self surfaces. However, inappropriate activation of the complement system can drive immune and inflammatory diseases [65]. The complement system plays a dual role in many conditions, including cancer [66]. The activated complement system can attack cancer cells; however, it can also participate in cancer progression via chronic inflammation. Inflammation represents the primary pathogenic feature of KS, and KSHV-infected cells establish the microenvironment to facilitate tumor growth and survival by producing proinflammatory and proangiogenic factors [51]. Complement activation may, therefore, contribute to the pathogenesis of KS. 

The formation of MAC on cell surfaces induces cell death by creating membrane pores. MAC-induced lysis is readily observed in the erythrocytes; however, this lysis appears to be very limited in nucleated eukaryotic cells. Although MAC-deposition was observed around the KSHV-infected cells in KS tissue, cell lysis was not prominent [11]. In cultured KSHV-infected human endothelial cells, MAC formation induced 10–20% cell death within 0.5–2 h of activation. However, cell death by complement activation did not progress, and cells ultimately recovered from the complement attack. Similar phenomena have been reported in various cells, including human cancer cells, retinal pigment epithelium, lung epithelial cells, and endothelial cells [32,33,67,68,69]. A balance between the triggering of complement activation and the defense mechanism against complement activation determines the fate of the cells. Since the classical pathway depends on the concentration of antibodies, high in vitro antibody levels generally exhibit more potent effects on cells than those observed in the alternative pathway. We found that the alternative pathway is activated in various eukaryotic cells, which can cause cell injury; however, not all cells die as a result [11,50,69,70]. Hence, compared to the classical pathway, the alternative pathway appears to have limited capacity to generate a sufficient number of MAC to kill cells. 

**Figure 3:**
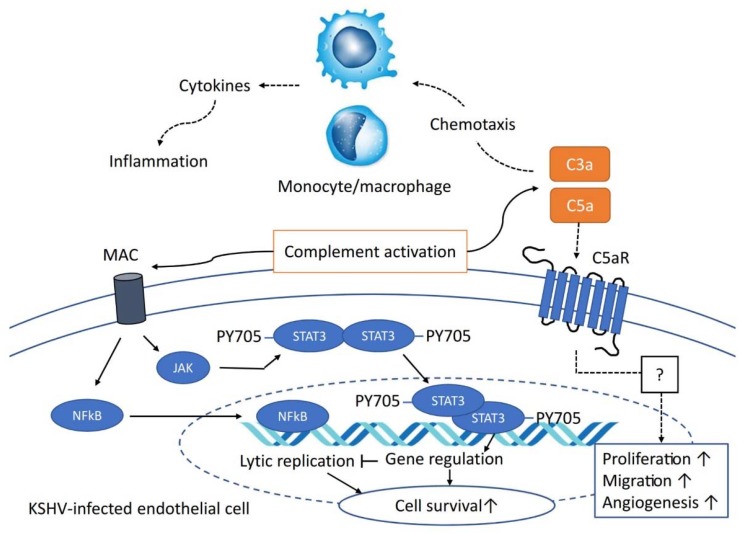
The role of the activated complement system in KSHV pathogenesis. The activated complement system induces the deposition of membrane attack complex (MAC) on the cell surface. Sublytic MAC activates the JAK-STAT3 pathway and NF-κB pathway in latently KSHV-infected human endothelial cells and de novo KSHV-infected human endothelial cells, respectively. Both activated pathways increase cell survival. Dotted lines from C3a and C5a indicate their putative roles in KSHV pathogenesis. C3a/C5a may serve as chemotactic factors for monocytes and macrophages inducing inflammatory cytokines. C5a may increase proliferation, migration, and angiogenesis through C5a–C5a receptor interaction.

Sublytic MAC can stimulate several cell types by calcium-influx-mediated signaling pathways, including the AKT, SRC, and ERK pathways [31,71]. A rapid rise in intracellular calcium concentrations caused by external calcium leakage through MAC pores activates various signaling molecules. Complement activation in the KSHV-infected endothelial cells increases phosphorylation of STAT3, which provides a survival benefit in growth-factor-free culture conditions [11]. Moreover, knockdown of both CD55 and CD59 in KSHV-infected TIME cells suppresses the phosphorylation of STAT3. Since MAC is the only complement factor inhibited by both CD55 and CD59, the STAT3 pathway is likely also affected by MAC formation [11]. Complement activation in PEL cells also confers a survival advantage; however, its underlying mechanism remains to be elucidated. KSHV induces lytic replication in HUVECs after infection, resulting in an increased number of dead cells. As shown in Section 4, de novo infection of HUVEC by KSHV induces the activation of the complement system. The formation of MAC does not kill the KSHV-infected HUVECs but rather increases their survival, which is due to the sublytic MAC-mediated activation of the NF-κB pathway, which subsequently suppresses the lytic replication of KSHV [49]. Collectively, the complement system confers survival benefits to KSHV-infected cells in myriad ways (Figure 3). 

During complement activation, C3a and C5a are released following cleavage of C3 and C5 protein, respectively. These fragments bind to specific receptors on several types of cells, including mast cells, triggering a non-IgE-mediated degranulation of histamine and other mediators related to allergic reactions [37]. Thus, their actions mimic anaphylaxis; accordingly, they are called anaphylatoxins. Anaphylatoxins can also induce IL-1 and IL-6 production, contributing to the systemic inflammatory response [72,73]. Human endothelial cells express both C3a and C5a receptors. The interaction of C3a/C5a with receptors on endothelial cells upregulates the expression of ICAM-1, increasing adhesion and stimulating extravasation into the tissues at the site where an antigen has triggered complement activation. A previous study showed that C5a enhanced cell proliferation, migration, and angiogenesis in human endothelial cells [74]. C5a also induces cell surface expression of adhesion molecules and complement receptors in macrophages and monocytes. Deposition of C3b and MAC were observed in KSHV-infected endothelial cells and PEL cells, which indicates that C3a and C5a can be released into the microenvironment. Hence, C3a and C5a may play crucial roles in KS pathogenesis. Chemotaxis and increased immune cell adhesion are the main pathological features of KS tissue. KSHV infection in endothelial cells has been shown to induce IL-6 production; however, anaphylatoxin may enhance inflammation by stimulating the production of IL-1, IL-6, and TNF-α (Figure 3). 

## 6. Conclusions and Future Perspectives

In summary, KSHV encodes KSHV ORF4 (which exhibits a complement regulatory function), which contributes to the evasion strategy of KSHV from complement attack. In spite of KSHV ORF4, the alternative complement pathway can be activated in KSHV-infected cells. While KSHV suppresses the cellular complement regulatory proteins to activate the complement system in latently infected cells, different mechanisms are involved in the de novo infection of KSHV. The activated complement system in KSHV-infected cells does not induce extensive cell lysis but appears to be exploited by KSHV for persistent infection in host cells.

Despite these findings, numerous unanswered questions remain. The biological role of KSHV ORF4 is not clear in KSHV-infected cells. As KSHV ORF4 primarily inhibits the classical pathway, its role in the inhibition of the alternative pathway in KSHV-infected cells appears to be limited. Based on the experimental evidence of complement activation in KS tissues, KSHV ORF4 may not completely inhibit the complement system. Since considerable MAC pore formation induces cell death, KSHV ORF4 may be able to suppress the classical pathway while supporting cell survival following the activation of the alternative pathway. However, further research is needed to fully elucidate the precise role of KSHV ORF4. It is also important to clarify the precise mechanisms of complement activation in KSHV-infected cells. While suppression of complement regulatory proteins such as CD55 and CD59 in latent infection causes the activation of the alternative pathway, the expression of these complement regulatory factors is not suppressed in de novo KSHV infection. Hence, the mechanism for controlling the complement regulatory proteins in KSHV-infected cells remains to be elucidated. Moreover, further analysis using an improved experimental model is required to uncover the complement activation mechanisms in de novo infection, including the precise EV factors associated with complement activation.

Finally, the complement system plays critical roles in the immune response as well as in various biological processes in the microenvironment. Given that the microenvironment is essential to the pathogenesis of KSHV, the complement system is also likely to be closely related. Although a function for the sublytic MAC was previously suggested in KSHV-infected cells [11,49], the specific functions of the complement system in vivo remain far from fully elucidated. Future investigation of these questions may provide novel insights into the complement system in KSHV pathogenesis.
